# Value Propositions of Public Adult Hearing Rehabilitation in Denmark

**DOI:** 10.3390/audiolres13020023

**Published:** 2023-04-12

**Authors:** Katja Lund, Rodrigo Ordoñez, Jens Bo Nielsen, Stine Christiansen, Sabina Storbjerg Houmøller, Jesper Hvass Schmidt, Michael Gaihede, Dorte Hammershøi

**Affiliations:** 1 Department of Electronic Systems, Aalborg University, 9220 Aalborg, Denmark; 2 Department of Otorhinolaryngology, Head & Neck Surgery and Audiology, Aalborg Universitety Hospital, 9000 Aalborg, Denmark; 3 Department of Health Technology, Technical University of Denmark, 2800 Kgs. Lyngby, Denmark; 4 Research Unit for ORL—Head & Neck Surgery, 5230 Odense, Denmark; 5 OPEN—Odense Patient Data Explorative Network, Odense University Hospital, 5230 Odense, Denmark

**Keywords:** value propositions, qualitative analysis, hearing aids, hearing-loss rehabilitation, probabilistic choice models, paired comparisons

## Abstract

**Objective:** To obtain and evaluate detailed descriptions of potential value propositions as seen by adults undergoing hearing rehabilitation with hearing aids. **Design:** Semi-structured interviews with patients and audiologists, a literature search, and the inclusion of domain knowledge from experts and scientists were used to derive value propositions. A two-alternative forced-choice paradigm and probabilistic choice models were used to investigate hearing aid users’ preferences for the value propositions through an online platform. **Study sample:** Twelve hearing aid users (mean age 70, range 59–70) and eleven clinicians were interviewed. A total of 173 experienced hearing aid users evaluated the value propositions. **Results:** Twenty-nine value propositions as described by patients, clinicians, and hearing care experts where identified, from which twenty-one value propositions were evaluated. Results of the pair-wise evaluation method show that the value propositions judged to be the most important for the hearing aid users were: “13. To solve the hearing problem you have”, “09. Thorough diagnosis of the hearing”, and “16. The hearing aid solution is adapted to individual needs”, which are related to finding the correct hearing solution and to be considered in the process. The value propositions judged to be least important were: “04 Next of kin and others involved in the process”, “26. To be in the same room as the practitioner”, and “29. The practitioner’s human characteristics”, related to the involvement of others in the process and the proximity and personal manner of the practitioners.

## 1. Introduction

Consumer value has moved from a goods-dominant logic, in which value is embedded in the products themselves, to a more service-dominant logic, where focus on consumer experience increases [1,2]. In the service-dominant logic, value is not defined by one actor alone, such as a manufacturer, but is co-created and assessed by several stakeholders, including the customer. Moreover, value is perceived more as an outcome of the interaction and activities during the interaction with a product rather than embedded in the product itself [2,3,4].

In the service-dominant logic, a company may offer (rather than deliver) value propositions, as value is said to be co-created through interaction and defined by the context of the consumer [1]. The concept of value propositions is, therefore, not a simple construct as different products enable different types of interactions, similar to how consumers interact in different ways with a product or service.

Consumer value within audiology is often measured in outcome or satisfaction. The outcome of hearing aid (HA) use is a multi-dimensional construct [5]; therefore, successful use depends on several relevant aspects. HA outcomes are often assessed through validated questionnaires focusing on elements such as sound quality, audibility, and speech intelligibility. In a recent Swedish study [6], IOI-HA questionnaires and additional questions were sent to HA users 3–6 months after fitting to measure the outcome. The results showed that bilateral fitting and HA experience positively affect the reported outcome. There was a significant correlation between the perceived service level of the dispensing clinic and the technical functionality of the HA. In another study, Laplante-Lévesque et al. [7] used focus groups to generate data on the views of HA users and audiologists in optimal HA use. A qualitative content analysis showed that elements such as a patient’s access to information, adjustments, audiologic practice, and profession, and benefits and limitations of the HA are determinants for optimal HA use. Recently, a study based on online consumer reviews by HA users, Bennett et al. [8], found that reviews focused mainly on device acquisition and device use. The former represents the price, manufacturer, physical fit, and management of the device. The latter represents the benefit of hearing in noise and technological features, such as options for streaming sound, mobile phone integration, and self-adjustment. In an earlier study, Bennett et al. [9] explored HA users’ and clinicians’ opinions on the skills required for HA management and overall success with HA treatment. Through concept mapping techniques (concept mapping is a mixed method combining qualitative approaches to data collection with quantitative data analyses to create visual maps of the participants’ opinions), six concepts were identified as important for HA management: “Advanced HA Knowledge”, “Daily HA Use”, “HA Maintenance and Repairs”, “Learning to Come to Terms with HA”, “Communication Strategies”, and “Working With Your Clinician”.

These studies, which all assess elements of value in the hearing rehabilitation process, apply different methods for data collection and analysis showing that different approaches lead to varying results with some overlapping themes. The aims of the present study are (1) to acquire a meaningful set of descriptions of value propositions in a public hearing rehabilitation context, and (2) to ask a large number of HA users to evaluate the value propositions in terms of importance for their rehabilitation process. First, through interviews with patients and audiologists and literature and domain knowledge from experts and scientists, a reduced list of value propositions was elaborated through a distillation process. Second, experienced HA users who did not participate in the interviews were asked to evaluate the value propositions through paired comparisons, in order to derive a ratio scale for the value proposition preference.

## 2. Materials and Methods—Harvesting Value Propositions

### 2.1. Participants

Twenty-three interviews were carried out from March to May 2021. Twelve HA users were interviewed after the fitting or adjustments of their HAs in a Danish public hearing clinic, either in Odense or Aalborg. Nine men and three women aged 59–74 years (average 70) were interviewed. Two women and one man had been participating in the BEAR-project (www.bear-hearing.dk, accessed on 11 April 2023) and had, thus, been fitted using the BEAR-fitting rationale [10]. The HA users interviewed were both new and experienced, as they were likely to have different views on the value elements related to HA uptake and use, and as such, represent diversity with respect to the different stages of the rehabilitation process. They also represent the typical age group that is referred to clinics in the Danish public health system. All interviews were carried out online via a webcam due to COVID-19 restrictions at the time.

Interviews with eleven clinicians were made over the telephone or web-based using a webcam.Eight women and three men working in the audiology departments at Odense University Hospital or Aalborg University Hospital participated. Three of the practitioners were also working on the BEAR project by applying new tests and fitting strategies.

The decision to include BEAR patients and BEAR staff was based on the belief that it would have been difficult for the HA users to imagine what one had not experienced and, thus, imagine what could have increased the value in the process. The BEAR-patients and the staff all received or gave treatments that—to a varying extent—differed from the traditional treatment in the clinics. In this way, the view was broadened on what may have been experienced as valuable to the HA user in a hearing rehabilitation trajectory.

### 2.2. Data Collection

The interviews were semi-structured, and the questioning technique was Socratic. In a Socratic dialogue, there are no closed questions, only curiosity and encouraging questions that support the interviewee in holding the subject in focus [11]. The interviewer is empathetic, provides emotional feedback, and frequently summarises. In the Socratic interview method, the interviewer facilitates the interviewee’s ability to express their points of view, past experiences that describe value elements, as well as wishes and dreams for the ’perfect’ hearing rehabilitation experience described from a value-based perspective. Each interview lasted between 15 and 30 min.

A theoretical approach to value propositions, according to Osterwalder et al. [12], involves describing three main aspects: (1) Tasks or challenges that patients need to have resolved when they contact the clinic; (2) Pains or the obstacles and problems the patients experience while attempting to have the task resolved, such as challenges related to the hearing rehabilitation process; (3) Gains or the improvements experienced when the task is resolved, such as the benefit of wearing a hearing aid. These three elements were kept in mind by the interviewer throughout the interviews. New interviews were scheduled and conducted until new insights became increasingly rare (saturation).

### 2.3. Inductive Thematic Analysis

The interviews were transcribed and imported into NVivo, (NVivo Qualitative Data Analysis software, Version 12, 2018. Lumivero, 1331 17th Street, Suite 404, Denver CO 80202, USA). All sentences potentially expressing value were extracted from the data. Several sentences expressed similar experiences and value elements. However, all sentences were included in the distillation process to avoid leaving out important value elements.

During the spring and summer of 2021, eight online workshops, each lasting approximately one hour, were completed to extract and group value elements from the sentences. The workgroup involved in this part of the process consisted of three researchers from the Department of Electronic Systems at Aalborg University (AAU) (R.O., D.H., and K.L.) and one researcher from the Department of Health Technology at the Technical University of Denmark (DTU) (J.B.N.). A structured process took place in which the sentences were read out loud, discussed, labelled, and grouped. A full-day workshop where all workgroup members participated physically was arranged to complete the first level of thematic grouping.

The goal of the distillation process was to reach a reduced number of value propositions. Each value proposition should have a fulfilling, but short, description of the value elements included and a descriptive headline.

### 2.4. Deductive Thematic Analysis

To ensure that the harvesting process did not leave out critical value elements known in the field, a literature search was made on the words ‘hearing aid’ AND ‘audiology’ AND ‘outcome’ AND ‘value’ AND ‘satisfaction’. Searches combining the words above in smaller groups and pairs, as well as including the words ‘up-take’, ‘success’, ‘self-report’, ‘experience’, ‘use’, ‘value proposition’, and ‘consumer review’ were made. The search returned a large number of studies identifying elements relevant to successful HA use, some of which were mentioned in the introduction. The studies were continuously compared with the value labels found in the interviews to ensure full coverage of potential value elements.

A workshop based on participatory design thinking was planned and completed in late summer 2021. The value propositions extracted by the workgroup were treated further at this stage. Twelve BEAR project members participated, including two facilitators from the value propositions workgroup (R.O. and K.L.). The aim of the workshop involved BEAR participants from all areas (industry, academia, hospitals) to have their expert views on patient value to further develop and discuss the value propositions.

The workshop lasted an hour, in which the enumerated value proposition headlines were projected on a screen and the associated descriptions were made available for the participants to read.

The participants were asked to:

1. Read the headlines and the detailed descriptions of the value propositions to consider if any themes were missing from the headlines;
2. Consider merging value propositions;
3. Consider the wording of each value proposition;
4. Consider the degree of detail needed.

The facilitators helped during the process by engaging in discussions if invited by the participants. The workshop yielded relevant suggestions for each of the bullet points above.

As a final step in the distillation process, all BEAR project members were asked to complete an online poll in which each member received five votes to indicate which value propositions they considered to be the most important from their point of view but with a clear focus on patient value. All value propositions identified were listed, and 17 BEAR project members completed the poll. The results from this poll are referred to as the scientists’ classification.

## 3. Results—Harvesting Value Propositions

### 3.1. Value Propositions List

The workshop-based distillation and grouping process resulted in twenty-nine value propositions. The value proposition headlines and their corresponding narratives are presented in Table 1, Table 2, Table 3 and Table 4. The tables include both the original Danish formulation and an English translation. *Italic* text in each cell denotes the headline, and the normal text is the corresponding narrative.

### 3.2. Selection of Value Propositions for Paired Comparisons

To be included in the set of value propositions for the evaluation (paired comparison); the following four inclusion criteria were used:

A Will the subject be well-elucidated by being included in the test?
B Is the subject a known and frequent factor in the literature and/or in general?
C Is the subject represented in the scientists’ classification?
D Is the subject represented in interview data?

**Figure 1:**
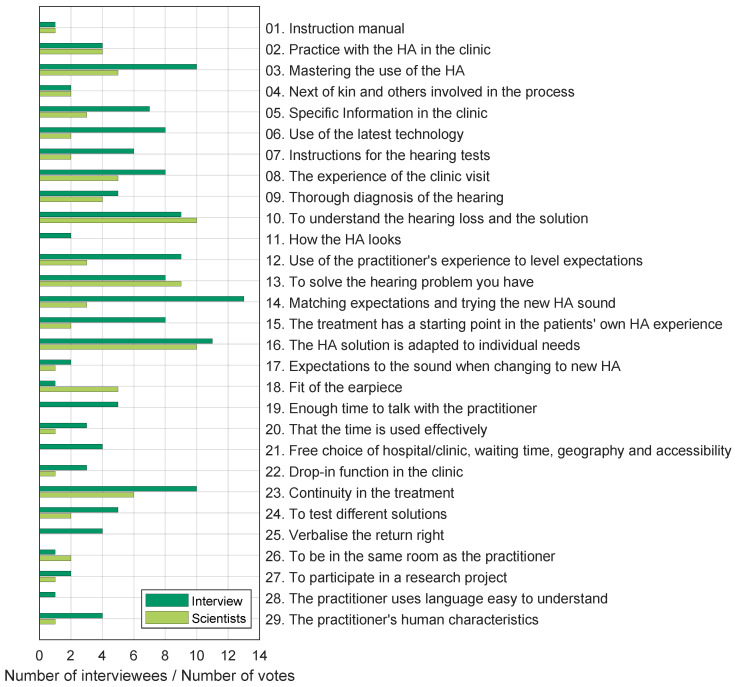
Count of how many interviewees mentioned a given value proposition (dark green bars). Number of votes given to each value proposition by the participants of the scientists’ classification (light green bars). The twenty-nine value labels from the distillation process are listed on the *y*-axis.

It was decided to include a given value proposition if three or more of the above criteria were confirmed. The interview data were revisited after having completed the distillation process to count the number of interviewees mentioning each value proposition (see Section 2). The value propositions that were mentioned by most patients and practitioners during the interviews were “14. Matching expectations and trying the new HA sound”, which was mentioned by 13 interviewees—8 were practitioners and 5 were patients; “16. The HA solution is adapted to individual needs”, was mentioned by 11 (mainly) practitioners and only 1 patient; “03. Mastering the use of the HA” and “23. Continuity in treatment” were both mentioned 10 times by 5 practitioners and 5 patients. All value labels were mentioned by at least 1 interviewee. In terms of the scientists’ classification, the most voted value propositions were “10. To understand the hearing loss and the solution” and “16. The HA solution is adapted to individual needs”, each receiving 10 votes; “13. To solve the hearing problem you have” received 9 votes, while “23. Continuity in the treatment” received 6 votes. A few value propositions did not receive any votes. This does not mean that they were perceived as unimportant, but as each scientist only received 5 votes, an independent prioritisation was made by each scientist. The results of the count of the value proposition from the interview data and the scientists’ classification are shown in Figure 1.

Based on the inclusion criteria, a list of 21 value propositions was reached. Inclusion criteria A and B were discussed in the workgroup and each value proposition was evaluated separately. Sixteen value propositions fulfilled all four criteria, five fulfilled three criteria, all of which fulfilled criteria A and B; two of these five were also in the scientists’ classification (criterion C), and the remaining three were represented in the interview data (criterion D). The chosen value propositions and their associated narratives are highlighted in Table 1, Table 2, Table 3 and Table 4 using bold fonts for the numbers and headlines.

## 4. Methods and Materials—Evaluation through Paired Comparisons

The 21 chosen value propositions and their narratives were used in a 2-alternative forced-choice experiment where HA users could choose the preferred value proposition from all unique pairs.

### 4.1. Participants

An invitation to participate in the study was sent to approximately 800 HA users who underwent HA fitting in Denmark between January 2017 and January 2018 as part of the cohort reported by Houmøller et al. [13]. None of them participated in the interviews described in Section 2. The invitation contained a description of the study, instructions, a web address to the response system, and a personal user ID and password. Data collection was carried out in March 2022, and participants were asked to complete the study within two weeks. A total of 395 HA users logged into the system and 173 completed the study.

### 4.2. Data Collection

Data were collected through a web page programmed in HTML, CSS, JavaScript, and PhP, hosted on an Apache web server running on a virtual Linux server at Aalborg University’s network. All collected data were anonymised and saved as plain text files on the university’s servers. After successfully logging in to the system, respondents were presented with two value propositions and their corresponding narratives in two large ’buttons’ placed side by side, underneath the question: “What is more true?”. To make a choice, the respondents pressed one of the two buttons and a green square with a white check mark appeared on the selected button. Once the choice was made, a “Next” ’button’ became active, allowing the respondent to continue to the next comparison. The respondents were also allowed to review and/or change the previous answer given by pressing the “Previous” ’button’.

All unique pairs of value propositions were presented to the participants, with each value proposition presented an equal number of times on each side of the screen (left or right response ’buttons’). The order of the comparisons was randomised between respondents. Each participant who completed the study with N=21 value propositions made a total of N(N−1)/2=210 comparisons.

### 4.3. Response Reliability

The results are first analysed for within-subject consistency by counting the number of circular triads for each respondent. For each set of three different value propositions or triads (a≠b≠c, where a,b,c=1,2,⋯,N), a circular triad occurs when *a* is chosen over *b*, *b* is chosen over *c*, but *c* is chosen over *a*. Using the number of circular triads, two inclusion criteria are considered: (1) Using the procedure originally described by Kendall and Smith [14] and used by [15], a $\chi^{2}$ test statistic can be derived from the number of circular triads and those expected by chance alone. This method is used to identify respondents who are answering better than chance; (2) The number of observed circular triads for each respondent can also be compared to the maximum number of possible circular triads given by the number of stimuli being compared. According to Kendall and Smith [14] and for *N* stimuli, this becomes $(N^{3}-N)/24$. An arbitrary number of circular triads can then be used as an inclusion criterion, in the present case, this is set to 30% of circular triads (or 70% consistent judgement).

### 4.4. Preference Model and Ratio Scale

To build a ratio scale based on preference judgements, a compound preference matrix was used. It contained the sum of all respondents’ preferences for each comparison as input to the binary probabilistic choice model suggested by Bradley and Terry [16] and the choice axioms proposed by Luce [17], also known as the *BTL* model. If the preference judgements are independent of irrelevant alternatives, a ratio scale can be determined that describes the probability of choosing one alternative over others. However, this condition can be violated for stimuli that are not easily comparable (i.e., because stimuli are perceived as similar or equal) or because they are compared based on different perceptual aspects (i.e., not a unidimensional precept). To cope with the problem of stimulus similarity, Tversky [18] proposed the family of models called elimination by aspects (EBA), in which the choices are based on specific stimuli attributes. If a given pair of stimuli share the same attributes, different aspects of the stimuli will drive the preference judgement, ignoring common attributes in the decision process. If all stimuli in question do not have common attributes, then the EBA model reduces to the BTL model. A specific case of the EBA model that considers a hierarchical decision strategy, referred to as preference trees (PT), can manage stimuli that are related by one or more attributes [19]. The advantage of EBA and PT models is that they can deal with complex decision criteria (at the cost of additional model parameters). For each common aspect or hierarchical relationship of the stimuli, one additional parameter is introduced in the model.

In order to identify the additional parameters for the PT and EBA models that represent relationships and common aspects among the value propositions, four members of the research team (R.O., K.L., S.S.H., S.C.) developed six overall categories describing specific aspects of the value propositions; they independently assigned each value proposition to one or more of these categories. Based on this input, a complex probabilistic choice model with eleven additional parameters was developed and applied to the data following the procedure outlined in Wickelmaier and Schmid [20]. Through an iterative process, model parameters that did not improve the model fit were removed, and the simplified model was applied until a model that best described the data was found. The comparison between the models was based on the number of model parameters and the ratio between the maximum likelihood estimates of a given model and that of a saturated model (with one parameter for each comparison N2). This ratio is approximately $\chi^{2}$-distributed with N2−(N−1+C) degrees of freedom, where *N* is the number of stimuli and *C* is the number of extra parameters added to the simple BTL model with one parameter for each stimulus. The models were rejected if the significance level was less than 10% (p<0.1).

## 5. Results—Evaluation through Paired Comparisons

**Figure 2:**
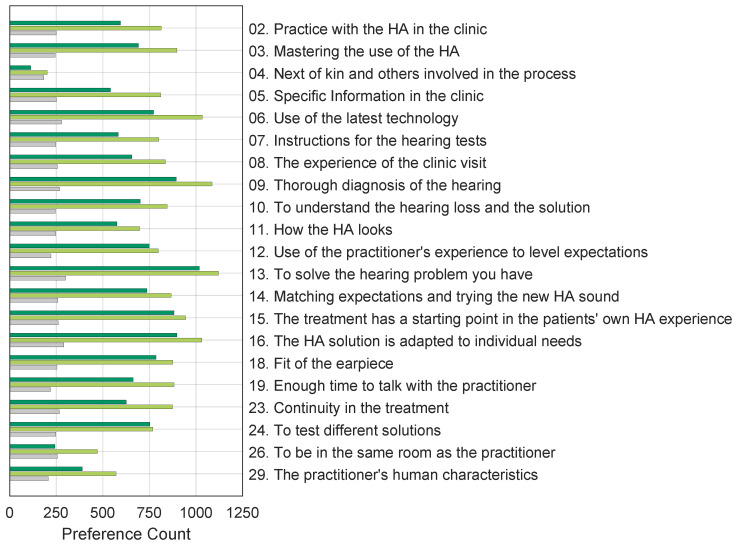
Number of times the 173 respondents chose each value proposition over the alternatives. Each bar indicates the number of responses following the different inclusion criteria. The bottom bars (grey) represent the contributions from respondents who had a response close to or worse than chance. The middle bars (light green) show the preference counts of the respondents who were better than chance but had >30% circular triads, and the top bars (dark green) show the preferences of respondents who had <30% circular triads.

Figure 2 shows the preference counts for all respondents, indicating the contribution of each of the three inclusion criteria described in Section 4.3. It is interesting to note that respondents who performed better than chance (top and middle bars in green colours) have a similar pattern of choices showing strong preferences for value propositions “06. Use of the latest technology”, “09. Thorough diagnosis of the hearing”, and “13. To solve the hearing problem you have”, and low preferences for value propositions, i.e., “04. Next of kin and others involved in the process”, “26. To be in the same room as the practitioner”, and “29. The practitioner’s human characteristics”, while the respondents who performed worse than chance (bottom bars in grey colours) have no clear preferences.

The categories developed to derive EBA and PT models, described in Section 4.4, are (1) “Technology and practice”: use of the HA, as well as the technology used in the HA and in the audiological assessment; (2) “Other people”: aspects relating to other people that can be, or are involved in the rehabilitation process; (3) “Information and communication”: aspects relating to the information received during the process and the communication methods used; (4) “Good experiences”: relating to positive or empowering experiences during the process; (5) “Individualised solutions and comfort”: aspects relating to individual needs, choices, and preferences that are taken into account during the process; and (6) “Time used”; the aspects related to the time used in different aspects of the process. These categories are not meant to describe the specific value propositions. They are meant to identify commonalities and relationships between the value propositions. For example, value propositions “02. Practice with the HA in the clinic” and “03. Mastering the use of the HA” have the common aspect of becoming accustomed to the HA and the technology in use. In the same manner, value propositions “06. Use of the latest technology” and “24. To test different solutions” focus on the technologies used in the rehabilitation process, so these four value propositions were grouped under category (1) “Technology and Practice”. The resulting model parameters obtained from the iterative process are summarised in Table 5. All three models have 21 parameters representing the stimuli; the EBA and PT models additionally have three parameters each, which are related to the specific value propositions given in the table.

**Figure 3:**
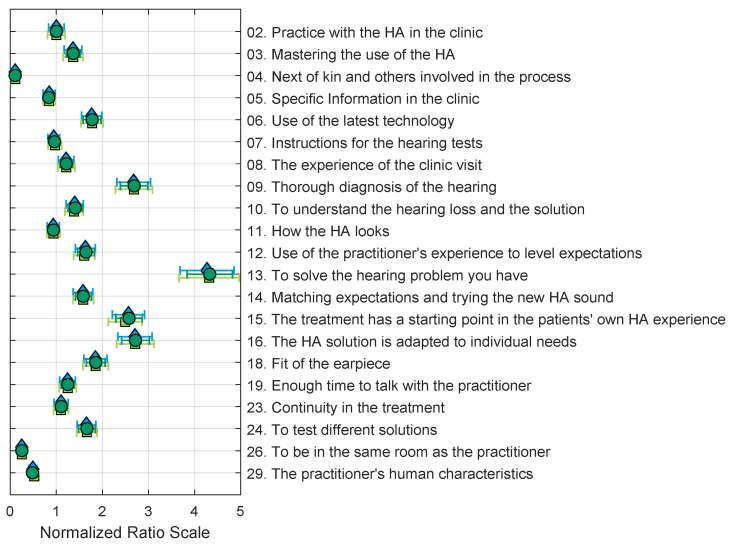
Normalised ratio scales derived with BTL (dark green circles), PT (blue diamonds), and EBA (light green squares) models using the 66 subjects with less than 30% circular triads (inclusion criterion 2). All estimates and standard errors are normalised by the estimate of value proposition 02. The goodness of fit of the models compared to a saturated model (see Section 4.4) are BTL: $\chi^{2}\left(190\right)=170.89$, p=0.83662; PT: $\chi^{2}\left(187\right)=169.26$, p=0.81934; EBA: $\chi^{2}\left(187\right)=166.96$, p=0.85099.

The normalised ratio scales resulting from the BTL, PT, and EBA models applied to the data of the 66 most consistent respondents (inclusion criteria 2 from Section 4.3) are shown in Figure 3. The estimates in the figure are normalised by the estimate of the value proposition “02. Practice with the HA in the clinic”, so that the scale value for this value proposition is one.

**Figure 4:**
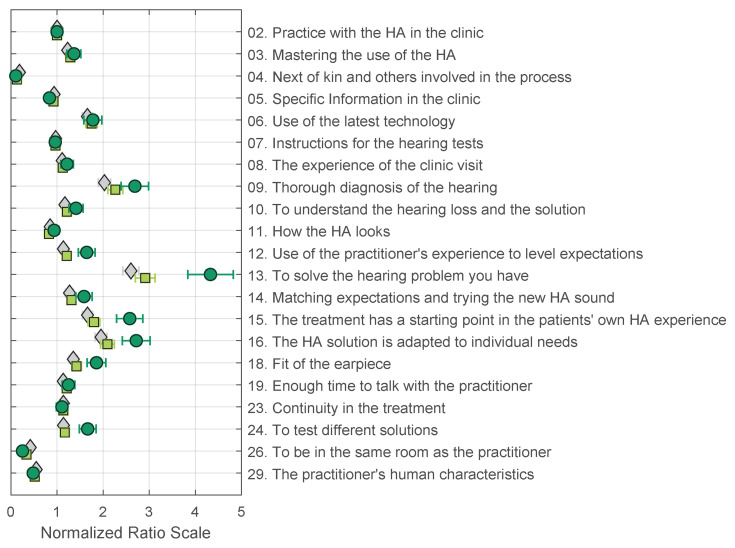
Normalised ratio scales for the BTL model derived using three inclusion criteria: All data from 173 respondents (grey diamonds); better than chance from 148 respondents (light-green squares); and <30% circular triads from 66 respondents (dark-green circles). All estimates and standard errors are normalised by the estimate of value proposition 02. The goodness of fit of the models compared to a saturated model (see Section 4.4) are as follows: all data (173): $\chi^{2}\left(190\right)=395.88$, p=0.0000; better than chance (148): $\chi^{2}\left(190\right)=268.13$, p=0.00016; <30% circular triads (66): $\chi^{2}\left(190\right)=170.89$, p=0.83662.

An analysis of the resulting estimate values, as well as all model parameter values, reveals that the additional PT and EBA parameters do not improve the resulting estimates, and are not statistically different from the BTL model. Comparing the BTL model fit using the different inclusion criteria shows that the model cannot account for respondents with more circular triads. This can be seen in Figure 4.

For inclusion criterion 2, a total of 54 weak stochastic transitivity (WST) violations were found. These violations were found for all value propositions except for value propositions “04. Next of kin and others involved in the process”, “13. To solve the hearing problem you have”, “26. To be in the same room as the practitioner”, and “29. The practitioner’s human characteristics”. This was also the case for inclusion criteria 1. Figure 4 shows that the general shape of the preference scales is similar for the different inclusion criteria; the main difference is that the extreme values are more pronounced. This means that when there is less noise in the data (less stochastic transitivity problems), a clear difference can be observed between value propositions that are desired (09, 13, and 16) and the ones that are not (04, 26, and 29). Another relevant observation is that the additional PT and EBA parameters tend to disappear with more consistent subjects, reducing the models to their simplest forms with one parameter for each value proposition, i.e., the BTL model. As seen in Figure 3, the three models yield virtually identical estimates for all value propositions.

## 6. Discussion

The value propositions judged to be most important by respondents were: “13. To solve the hearing problem you have”, “09. Thorough diagnosis of the hearing”, “16. The HA solution is adapted to individual needs”, and “15. The treatment has a starting point in the patients’ own hearing aid experience”. The narratives for these preferred value propositions include “...a good correspondence between hearing tests and the patient’s hearing needs and challenges that they experience daily”, “...that, the practitioner understands the patient’s hearing problem, and attempts to solve the problem by approximating the listening situation that the patient describes and wants to change”, “...that, the practitioner has insight into the patient’s daily routine to be able to adapt the HA for the patient’s special needs”, and “As a patient, you should have the opportunity to talk to the practitioner at a point when you have obtained some experience with the HA”. This indicates that the main value element that the respondents were concerned with was in meeting the patients’ daily individual hearing needs; it is important that both clinical hearing tests and hearing care professionals tap into this during the full process of the hearing rehabilitation. From the first contact with the clinic—or earlier—to the process of diagnosis and treatment in the clinic and follow-up after a period of HA use, it is important that the patients are able to communicate the challenges they face in different situations in their daily lives. However, based on numerous theoretical approaches to communication, we know that messages sent and messages received are not identical. With hearing loss involved, the challenges are even more pronounced. In Lund and Rosenstand [21], the authors describe how the same information may be sent (coded) and received (decoded) in many ways. The received message is, thus, no more than a qualified guess. Therefore the context is taken into consideration in all communication. When hearing loss is involved, there is a risk that some of the auditory communication is lost and the context becomes even more important. Considering the results, it should be noted that the respondents had all participated in a clinical trial, where the current practice was mapped beyond the standards (real-ear measurements for documentation, pre- and post-fitting questionnaires, follow-ups, etc.) [13]. This may explain the importance attributed to value proposition “09. Thorough diagnosis of the hearing”, as all respondents went through an extensive hearing examination as part of the clinical trial.

The lowest rated value proposition is “04. Next of kin and involved in the process”. This value proposition is perhaps more individually anchored than the value related to having one’s individual hearing needs met and solved, which represents a more general value aspect in hearing rehabilitation. It is important to note that not all patients will have the same need for help from significant others and some may be able and willing to handle their hearing aids themselves. However, in current practice, accompanying persons are not always encouraged to participate, which may give the impression that they are insignificant in the rehabilitation process.

Other lower-rated value propositions are “26. To be in the same room as the practitioner” and “29. The practitioner’s human characteristics”. The detailed descriptions of these include “It has value in that, you as a patient are in the same physical room as the practitioner...”, which could indicate a larger degree of preparedness for e-Health initiatives than earlier among patients seeking help at an audiological clinic. Here, it is important to mention that the participating patients went through audiological testing in clinical environments as part of a clinical trial. The patients were placed in soundproof enclosures or controlled conditions, and in some cases, they were separated from the practitioner conducting the tests. Thus, it is also likely that “being in another room as the practitioner” was interpreted as an adjacent room in the clinic, and not as a remote location.

Other narratives describing the lowest rated value propositions support the idea of remote treatment, as it seems less important that “...the practitioner is accommodating so you as a patient experience good treatment and service”. This is in line with the description of the value proposition “26. To be in the same room as the practitioner” stating “...that the practitioner takes into consideration good communication so that you as a patient can hear and see the practitioner clearly under the entire process”. The above discussion about communication and context, as well as studies by Arlinger et al. [6], tell us that this is—despite lower ratings—important, but it may be secondary when compared to the value propositions that address having the individual hearing problem solved. The patients do not necessarily know the value of good communication, service level, or support from a partner, as it may be experienced as implicit in the process of reaching the primary goal. It should, however, be brought to mind that all 21 value propositions included in the test went through a process of selection, indicating that all, to some degree, are valuable in a public hearing rehabilitation context.

The discussion of the lowest and highest rated value propositions highlights the significance of hearing care professionals understanding the value elements of the process towards resolving individual hearing problems and addressing them throughout the journey. Patients are not expected to be experts in communication, hearing technology, or hearing tests, but they are experts in their own hearing experiences. In addition, the hearing care professional serves as a facilitator by combining the pedagogical and expert aspects of hearing care, providing patients with "tools" to express their individual experiences in as many ways as possible, and leading the process toward solving the hearing problem.

The paired-comparison method used to obtain preference judgements from respondents is traditionally not applied to this type of conceptual comparison. Instead, this method is typically used to compare short-duration stimuli presented simultaneously or in rapid succession to investigate quality or preference judgements (e.g., [15,22]). One consideration for this method is the number of stimuli to compare because the number of comparisons increases rapidly with the number of stimuli, N2. In the present study, a total of 21 value propositions were compared, giving a total of 210 unique comparisons. Out of a potential population of 800 respondents, 395 logged into the system and a total of 173 completed the 210 comparisons. No feedback was systematically collected from participants who opted out, but some of them reached out to the research team (through telephone or mail). From this limited feedback, it became clear that, for some respondents, the task was overwhelming and that the introduction letter and instructions sent through electronic post were not sufficiently clear for all possible participants. In-person instruction and more detailed explanations of the method are necessary to obtain a higher response rate.

The results presented in this study demonstrate that it is possible to formulate relevant value propositions for hearing rehabilitation, taking into consideration the current practices in the clinics, as well as the experiences and opinions of both patients and practitioners. These value propositions can be evaluated by hearing-aid (HA) users in a paired comparison paradigm, eliminating the need for a rating scale. In practical terms, the respondents have only two options to consider, and they are not asked to evaluate to what degree they agree/disagree with a given statement (as in the case of e.g., Likert scales), simplifying the task. Finally, probabilistic choice models present a clear statistical framework to verify and analyze responses in order to create a ratio scale based on the respondents’ preferred value propositions.

## 7. Conclusions

In the present study, patients and practitioners were given the opportunity to express their opinions about what creates value in the hearing rehabilitation process, as represented by the public part of the Danish system. A 2-step inductive and deductive theme-based analysis was used to identify 21 value propositions that were considered relevant to assess more rigorously in a larger patient population, which eventually consisted of 173 participants. Using a two-alternative forced-choice paradigm, the results demonstrated enough consistency in responses to provide a ratio scale that revealed the most and least valued propositions among the given choices. The results reveal that patients appreciate a proper examination of their hearing problem, thorough diagnosis and individualised solutions, as well as the use of the latest technology. The results further suggest that patients are less appreciative of the potential advantage of involving next of kin in the process, nor do they consider the practitioner’s human characteristics to be significant. A less clear-cut conclusion can be drawn regarding the significance of being in the same physical room as the practitioner (suggesting readiness for teleconsultation), as this might reflect a majority understanding of the need for doing hearing tests in sound-proofed rooms with supervision from the adjacent room, both at the site of the clinic.

These results are, at best, representative of the adult Danish population dominated by presbycusis patients attending service at public university hospitals (approximately 40% of Danish patients). This method can be applied to other patient populations and rehabilitation procedures to explore the diversity of appreciation among patients and professionals and weigh contextual and population factors.

## Figures and Tables

**Table 1:** List of value propositions (1–8). The value propositions that were included in the succeeding pairwise comparison are indicated in bold font, i.e., the headline and number.

Number	English Translation	Original Danish
01	*Instruction manual.* It has value in that the hearing aid comes with a good instruction manual (printed and/or digital), which illustrates how one does various things and what one goes through in the clinic. It has to have pictures, be easy to use, manageable, and intuitive.	*Brugsanvisning.* Det har værdi, at der følger en god brugsanvisning med høreapparaterne (trykt og/eller digital), som illustrerer, hvordan man gør forskellige ting, og som bliver gennemgået i klinikken. Der skal være billeder og den skal være nem, overskuelig og intuitiv.
**02**	***Practice with the hearing aid in the clinic.*** It has value in that, as a patient, you have the time and possibility to practice putting the hearing aid on and taking it off, and changing the filters, batteries, etc., while there is a competent person who can help. In the clinic, there should be the possibility to change programs and adjust the level if the hearing aid has such functions.	***Øvelser med høreapparatet på klinikken.*** Det har værdi, at man som patient får tid og mulighed for at øve sig i at tage høreapparaterne af og på, skifte filtre og batterier og lignende, mens der er en fagperson, der kan hjælpe. I klinikken skal man også have mulighed for at prøve at skifte programmer og regulere på lydstyrken, hvis høreapparatet har den funktion.
**03**	***Mastering the use of the hearing aid.*** It has value in that, as a patient, you feel able to handle the hearing aid on your own without feeling unsure or handicapped, because it is too small or difficult to manage. Even though the hearing aid is small and advanced, it should be easy to operate and cover the individual’s everyday hearing needs.	***Mestring ift. håndtering af høreapparatet.*** Det har værdi, at man som patient føler, at man kan håndtere høreaparatet på egen hånd uden at føle sig usikker eller handicappet, fordi det er for småt eller for svært at håndtere. Selvom høreapparatet er lille og avanceret, skal det være let at betjene og dække hverdagens individuelle hørebehov.
**04**	***Next of kin and others involved in the process.*** It has value in that you receive help to handle the hearing aid when at home. It can, for example, be a caregiver or a next of kin that has been with you to the hearing clinic, and can support you in remembering what was said, as well as help explain the hearing situation to the hearing care professional.	***Pårørende og andre med i processen.*** Det har værdi, at man få hjælp til håndtering af høreapparatet, når man er hjemme. Det kan for eksempel være hjælp fra en hjemmehjælper eller en pårørende, der har været med på høreklinikken, og som kan støtte patienten i at huske, hvad der bliver sagt, samt forklare høresituationen til behandleren.
**05**	***Specific Information in the clinic.*** It has value in that, as a patient, you receive sufficient (yet not too much) information at the clinic. The information should be oriented toward the needs of the patient. Important information should be repeated, and it should be cohesive with the written material that is taken home. The process in the clinic should be clearly explained from the start, so one can concentrate on one thing at a time, without having to speculate about what the next step is.	***Målrettet information på klinikken.*** Det har værdi, at man som patient modtager tilstrækkelig, men heller ikke for meget, information på klinikken. Informationen skal være målrettet til patientens behov. Vigtig information skal gentages og hænge godt sammen med den skriftlige information, man får med hjem. Forløbet i klinikken skal være tydeligt forklaret fra start, så man kan koncentrere sig om én ting ad gangen uden at spekulere på, hvad næste skridt bliver.
**06**	***Use of the latest technology.*** It has value in that the most advanced test methods are used for hearing tests. The hearing aids should use the latest technology and should be able to connect to the patient’s telephone and other equipment used every day. The batteries in the hearing aid should preferably be rechargeable so there is no need to change the batteries.	***Anvendelse af ny teknologi.*** Det har værdi, at der under høreprøverne anvendes avancerede testmetoder. Høreapparaterne skal anvende den nyeste teknologi og skal kunne kobles op til patientens telefon og andre apparater i hverdagen. Batterierne i høreapparaterne skal helst være genopladelige, så man ikke skal skifte batterier.
**07**	***Instructions for the hearing tests.*** It has value in that instructions for the hearing tests are clear and easy to understand and the practitioner explains to the patient (you) what you will experience so that you feel sure about the task. The practitioner should pay attention to you, so, if necessary, test instructions are repeated, and the objective of the test is explained.	***Instruktion under høreprøverne.*** Det har værdi, at der er tydelig og let forståelig instruktion under høreprøverne, og at behandleren forklarer, hvad patienten kommer til at opleve, så man føler sig sikker på opgaven. Behandleren skal være opmærksom på patienten, så instruktioner om nødvendigt gentages og formålet med testen forklares.
**08**	***The experience of the clinic visit.*** It has value in that the visit to the clinic is a good experience for the patient (you). You should feel comfortable so that it will be a positive experience to receive the hearing aid. You should not feel that you have failed during the hearing test. You should feel that you can operate the hearing aid when you leave the clinic and that you are satisfied with the sound in the hearing aid.	***Oplevelsen af klinikbesøget.*** Det har værdi, at besøget på høreklinikken er en god oplevelse for patienten. Man skal føle sig godt tilpas, så det opleves positivt at få høreapparater. Man må ikke føle, at man har fejlet i høreprøverne. Man skal føle, at man kan betjene høreapparaterne, når man går fra klinikken, og man skal være tilfreds med lyden i høreapparaterne.

**Table 2:** List of value propositions (9–15). The value propositions that are included in the succeeding pairwise comparisons are indicated in bold font, i.e., the headline and number.

Number	English Translation	Original Danish
**09**	***Thorough diagnosis of the hearing.*** It has value in that the hearing tests are thorough and test different aspects of the hearing, so that the patient feels that there is good correspondence between the hearing tests, hearing needs, and challenges in everyday life, and that the patient feels that all aspects of the hearing loss are tested and clarified, and you can rely on the treatment offered.	***En omfattende undersøgelse af hørelsen.*** Det har værdi, at høreprøverne er grundige og tester forskellige aspekter af hørelsen så patienten føler, at der er god sammenhæng mellem høretests og de hørebehov og -udfordringer, der kan være i hverdagen. At man føler, at alle dele af høretabet bliver belyst, så man kan have tillid til den behandling, der bliver tilbudt.
**10**	***To understand the hearing loss and the solution.*** It has value in that the hearing care professional at the clinic provides the patient with a basic understanding about hearing loss, regarding, for example, range of speech, discrimination loss, and normal hearing, and that the patient (through different hearing tests) understands how hearing works, so that the patient understands existing solutions. The treatment should help the patients understand the significance of their hearing loss (for themselves and their next of kin).	***Forståelse af høreudfordringer og løsningsmuligheder.*** Det har værdi, at behandleren på klinikken giver patienten en grundlæggende forståelse af høretabet ift. fx taleområde, skelnetab og normal hørelse. At patienten via forskellige høretests får en forståelse af, hvordan hørelsen fungerer, så man forstår hvilke løsningsmuligheder, der er. Behandlingen skal hjælpe patienten med at forstå høretabets betydning, både for patienten selv og pårørende.
**11**	***How the hearing aid looks.*** It has value in that the hearing aid does not look too big (the outer part and the plug in the ear). In terms of looks, it is important that you feel comfortable wearing the hearing aid.	***Høreapparatets udseende.*** Det har værdi, at høreapparatet ikke ser for stort ud, hverken den ydre del eller proppen i øret. Rent udseendemæssig er det vigtig at man føler sig godt tilpas med at have sit høreapparat på.
**12**	***Use of the practitioner’s experiences to level expectations.*** It has value in that the practitioners can put themselves in the place of the patients and use their own experiences to assess what works well in view of the patient’s hearing loss. The practitioner must explain what the patient can realistically expect to receive from the hearing aid treatment. It should be clear what types of hearing aids can be chosen from, and what can be expected of them. It should be clear what the best choice is according to the practitioner.	***Anvendelse af behandlererfaring ved forventningsafstemning.*** Det har værdi, at behandleren kan sætte sig i patientens sted og bruge sin egen erfaring til at vurdere, hvad der virker godt ift. høretabet. Behandleren skal forklare, hvad man realistisk kan forvente at få ud af høreapparatbehandlingen. Det skal være tydeligt hvilke høreapparattyper, man kan vælge imellem, og hvad man kan forvente af dem. Det skal stå klart, hvad det bedste valg er set fra behandlerens synspunkt.
**13**	***To solve the hearing problem you have.*** It has value in that the practitioner understands the patient’s hearing problem, and attempts to solve the problem by imitating the listening situation that the patient describes and wants to have changed. It is important that the patients feel that there is a solution to their hearing problems, so that they can do some of the things they have not been able to do. Likewise, it has value in that the problems that the next of kin experience (with respect to the patient’s hearing loss) are understood and addressed.	***At man får løst det høreproblem, man har.*** Det har værdi, at behandleren forstår det høreproblem, patienten har, og forsøger at løse problemet ved at efterligne den lydsituation patienten beskriver og gerne vil have ændret på. Det er vigtigt at patienten føler, at der er en løsning på høreproblemet, så man kan nogle af de ting igen, man ikke har kunnet. Ligeledes har det værdi, at de problemer pårørende oplever ift. patientens hørelse bliver forstået og søgt løst.
**14**	***Matching expectations and trying the new hearing aid sound.*** It has value in that, as a patient, you can hear and experience how the sound changes with the new hearing aid. The patient will be allowed to test how the hearing aid sounds in the clinic and talk to practitioners through different stages of the process (regarding the new sound). The practitioner will explain that it can take time to become accustomed to the new sound and that not all sounds will be experienced as good as in the beginning.	***Forventningsafstemning og afprøvning af den nye høreapparatlyd.*** Det har værdi, at man som patient kan høre og opleve, hvordan lyden ændrer sig med de nye høreapparater. Patienten skal have lov til at afprøve lyden i klinikken og snakke med behandlere i forskellige dele af processen om den nye lyd. Behandleren skal forklare, at det kan tage tid at vænne sig til den nye lyd, og at ikke alle lyde vil opleves som gode til at starte med.
**15**	***The treatment has a starting point in the patient’s own hearing aid experience.*** It has value in that the treatment in the clinic is based on the patient’s own hearing aid experiences. You (the patient) should have the opportunity to talk to the practitioner at some point, after having some experience with the hearing aid. This is both immediately after you have been fitted with the hearing aid in the clinic, and at follow-up (after a few months).	***Behandlingen tager afsæt i patientens erfaringer med høreapparaterne.*** Det har værdi, at behandlingen i klinikken tager afsæt i patientens egne erfaringer med høreapparaterne. Man skal have mulighed for at snakke med behandleren på et tidspunkt, hvor man har gjort sig nogle erfaringer. Det er både lige efter, man har fået apparaterne på i klinikken, og ved opfølgning i klinikken efter nogle måneder.

**Table 3:** List of value propositions (16–23). The value propositions that were included in the succeeding pairwise comparisons are indicated in bold font, i.e., the headline and number.

Number	English Translation	Original Danish
**16**	***The hearing aid solution is adapted to individual needs.*** It has value in that the practitioner has insight into the patient’s everyday life (and, thus, will be able to adapt the hearing aid to any of the patient’s special needs). This can be, for example, related to adjusting the hearing aid to fit the patient’s environment and specific needs. It can also involve connecting the hearing aid to devices such as a telecoil, telephone, or television.	***Høreapparatløsningen tilpasses individuelle behov.*** Det har værdi, at behandleren har indsigt i patientens hverdag for at kunne tilpasse høreapparaterne til særlige behov. Det kan f.eks. dreje sig om en justering af høreapparaterne til patientens omgivelser og situation. Det kan også dreje sig om tilslutning af f.eks. teleslynge, telefon eller fjernsyn.
17	*Expectations to the sound when changing to new hearing aids.* It has value in that when changing hearing aids, the sound in the new hearing aid is as close as possible to the sound in the old hearing aid.	*Forventninger til nye lyde ved høreapparat-skift.* Det har værdi, at lyden i nye høreapparater ved skift er så lig lyden i de gamle apparater som mulig.
**18**	***Fit of the earpiece.*** It has value in that the plug fits well in the ear (and it is not annoying). It is possible to obtain a molded plug that fits, and if a plug is annoying, you can have a new one or the existing one can be fixed, so that the discomfort stops. Likewise, treatment is offered if there is eczema or other nuisances from the plug.	***Proppens pasform.*** Det har værdi, at proppen sidder godt i øret og ikke generer. At der er mulighed for at få lavet en støbt prop, der passer, og hvis en prop generer, at man kan få lavet en ny eller få den tilpasset, så generne ophører. Ligeledes, at der er tilbudt behandling, hvis der har været eksem eller andre gener skabt af proppen.
**19**	***Enough time to talk with the practitioner***. It has value in that the practitioners have plenty of time to talk about the treatment and explain possibilities and limitations with regard to hearing loss. The practitioner should have time to listen to and understand the patient’s hearing problems and pursue this to find the right solution. The patient shall have time and opportunity to ask questions.	***Tid til samtale.*** Det har værdi, at personalet har god tid til at snakke om behandlingen og forklare muligheder og begrænsninger ift. høretabet. Behandleren skal have tid til at lytte til og forstå patientens høreproblemer og følge det for at finde den rigtige løsning. Patienten skal have tid og lejlighed til at stille spørgsmål.
20	*The time is used effectively.* It has value in that the practitioner is well-prepared and has everything ready, so that the time spent with the practitioner is used effectively, without wasting time; it does not take too long when one has to go to the clinic (parking, driving, waiting to obtain an appointment, and waiting time in the clinic).	*At tiden bruges effektivt.* Det har værdi, at behandleren har forberedt sig og gjort alt klar, så tiden hos behandleren udnyttes effektivt, og der ikke er spildtid. At det ikke tager for lang tid, når man skal på klinikken (parkering, kørsel, ventetid for at få en tid på klinikken, ventetid i klinikken).
21	*Free choice of hospital/clinic, waiting time, geography, and accessibility.* It has value in that you are informed about the waiting time and alternatives, and you can choose the clinic yourself (for example, if it is a university hospital) when you need a new hearing aid; the clinic is close by and accessible, there are good parking options, and transport is free of charge, if necessary.	*Frit sygehus-/klinikvalg, ventetid, geografi og tilgængelighed.* Det har værdi, at man bliver oplyst om ventetider og alternativer og på den baggrund selv kan vælge høreklinikken, man vil til (fx om det er et universitetshospital), når man skal have nye høreapparater. At klinikken er tæt på og nem at komme til, at der er gode parkeringsmuligheder, og at man kan blive transporteret gratis dertil, hvis man har brug for det.
22	*Drop-in functions in the clinic.* It has/will have value in that you can drop in to the clinic without an appointment or with short notice, when you need an adjustment or to fix a small problem with the hearing aid.	*Drop-in funktion i klinikken.* Det har værdi/vil have værdi, at man kan droppe ind i klinikken uden tidsbestilling eller med kort varsel, når man har brug for at få lavet en justering eller for hjælp til et mindre problem med høreapparaterne.
**23**	***Continuity of the treatment.*** It has value in that there is continuity in the process before, during, and after the treatment in the clinic, as well as in the transition to the communication centre. This relation should be made clear to the patients when initiating the process. You (the patient) should have the same practitioner throughout the entire process, and the treatment should subsequently be at the same clinic. If you want, you you should be automatically invited to a follow-up and for hearing-aid renewal. Finally, the treatment shall be flexible and coherent with regard to individual needs, and you should be able to have earwax removed at the clinic.	***Sammenhæng i behandlingen.*** Det har værdi, at der er sammenhæng i forløbet under og efter behandlingen på klinikken og ved overgangen til kommunikationscenter. Sammenhængen skal tydeliggøres for patienten ved opstart af behandlingsforløbet. Man skal have samme behandler igennem hele forløbet, og efterfølgende skal behandlingen foregå på samme klinik. Hvis patienten ønsker det, bliver man automatisk indkaldt til en opfølgning og til fornyelse af høreapparaterne. Endelig skal behandlingen være fleksibel og sammenhængende ift. individuelle behov som fx at man kan få fjernet ørevoks på klinikken.

**Table 4:** List of value propositions (24–29). The value propositions that were included in the succeeding pairwise comparisons are indicated in bold font, i.e., the headline and number.

Number	English Translation	Original Danish
**24**	***To test different solutions.*** It has value in that you have the opportunity to test different types of hearing aids while at the clinic. In this way, you can experience the possibilities with respect to physical fit, the feeling of occlusion, etc., before deciding.	***Afprøvning af forskellige løsninger.*** Det har værdi, at man har mulighed for at afprøve forskellige typer høreapparater, mens man er på klinikken. Dermed kan man opleve mulighederne med hensyn til pasform, følelsen af indelukkethed osv., inden man beslutter sig.
25	*Verbalise the return right.* It has value in that the practitioner verbalises your right to come back after having tried the hearing aid for a while, and that you have the right to return it or exchange it at a follow-up, and that the device can be repaired free of charge.	*At man italesætter returretten.* Det har værdi, at behandleren italesætter retten til at henvende sig efter at have afprøvet høreapparatet i en periode, og at man har mulighed for at returnere det og/eller få det byttet ved en opfølgning, samt at det kan blive repareret gratis.
**26**	***To be in the same physical room as the practitioner.*** It has value in that you are in the same physical room as the practitioner, and the practitioner takes into consideration good communication so that you can hear and see each other clearly throughout the entire process.	***At være i samme fysiske rum som behandleren.*** Det har værdi, at man befinder sig i samme fysiske rum som behandleren, og at behandleren tænker over god kommunikation, så man under hele forløbet kan høre og se hinanden tydeligt.
27	*To participate in research projects.* It has value in regard to participating in research projects because, as a patient, you are examined more thoroughly and can receive a better hearing-aid solution; it can be interesting to participate in research because it is for a greater purpose.	*At deltage i forskningsprojekter.* Det har værdi at deltage i forskningsprojekter, fordi man som patient bliver undersøgt grundigere og dermed forventer at få en bedre høreapparatløsning. At det er interessant at deltage i forskning, fordi der er et større formål.
28	*The practitioner uses language that is easy to understand.* It has value in that the practitioner informs you in a language that is easy for you to comprehend without using too many technical terms.	*At behandleren bruger et letforståeligt sprog.* Det har værdi, at behandleren informerer på et sprog, der er til at forstå, hvor der ikke bruges for mange fagtermer.
**29**	***The practitioner’s human characteristics.*** It has value in that the practitioner is accommodating so that you experience good treatment and service. The tone should be warm and preferably humorous. The patient should feel that the practitioner is interested in helping. The practitioner should be polite, keep the patient informed about the process, be pedagogical in their approach, and show empathy.	***Behandlernes menneskelige egenskaber.*** Det har værdi, at behandleren er imødekommende, så man oplever en god behandling og service. Omgangstonen skal være varm og gerne humoristisk. Patienten skal opleve, at behandleren er interesseret i at hjælpe. Behandleren skal være høflig, holde patienten orienteret om forløbet, være pædagogisk i sin tilgang samt udvise empati.

**Table 5:** Overview of probabilistic choice model parameters.

Model	Parameters	Value Propositions
BTL	One for each stimulus	–
EBA	One for each stimulus	–
	Self-understanding / Confidence	02, 03, 04, 07, 08, 10
	Information and communication	05, 07, 10, 12, 15, 23, 29
	Technology and practice	02, 03, 06, 14, 24
PT	One for each stimulus	–
	Good communication with the practitioner	10, 12, 14
	Clear and concise information	05, 07
	Technology and practice	02, 03, 06, 24

## Data Availability

The data presented in this study are available on request from the corresponding authors. The data are not publicly available as the value propositions were elaborated from interviews carried out in Danish language, and as such, are only of limitted use for non-danish speakers in their original form. The translated headlines and narratives published here, represent the most accessible description of the value propositions obtained from the distillation process. As to the paired comparison data, a preference matrix as well as all invidual preferences from each respondent can be accessed upon request.

## Abbreviation

HA	hearing aid
